# Multiple myeloma: A closer look at one of its faces

**DOI:** 10.1002/jha2.1098

**Published:** 2025-01-22

**Authors:** Radu Chiriac, Zofia Gross

**Affiliations:** ^1^ Laboratory of Hematology Centre Hospitalier Lyon Sud Hospices Civils de Lyon Lyon France; ^2^ Department of Hematology Centre Hospitalier Lyon Sud Hospices Civils de Lyon Lyon France

**Keywords:** cytology, dysplasia, multiple myeloma, plasma cell

1

A 60‐year‐old man presented with worsening right‐sided facial paresthesia and persistent chin numbness, along with general deterioration and confusion for 3 weeks.

Laboratory investigations revealed hypercalcemia (3.5 mmol/L; normal: 2.2‒2.7 mmol/L) and elevated creatinine (330 µmol/L; normal: 65‒119 µmol/L). Serum protein electrophoresis showed no monoclonal band, while light chain analysis indicated free kappa light chains at 6.2 mg/L (normal: 3.3‒19.4 mg/L) and free lambda light chains at 8260 mg/L (normal: 5.7‒26 mg/L). Additionally, anemia (60 g/L) and thrombocytopenia (100 × 10⁹/L) were observed, with no abnormal circulating cells.

A whole‐body computed tomography scan revealed a mass in the right infratemporal fossa (Figure 1A, upper panel, asterisk) extending into the right maxillary sinus, causing lysis of its lateral wall and continuing into the pterygopalatine fossa, with potential involvement of the maxillary (V2) and mandibular (V3) nerves. A mass in the left cheek caused minimal lysis of the left maxilla (Figure 1A, bottom panel, asterisk). No other lytic lesions were observed.

**FIGURE 1:**
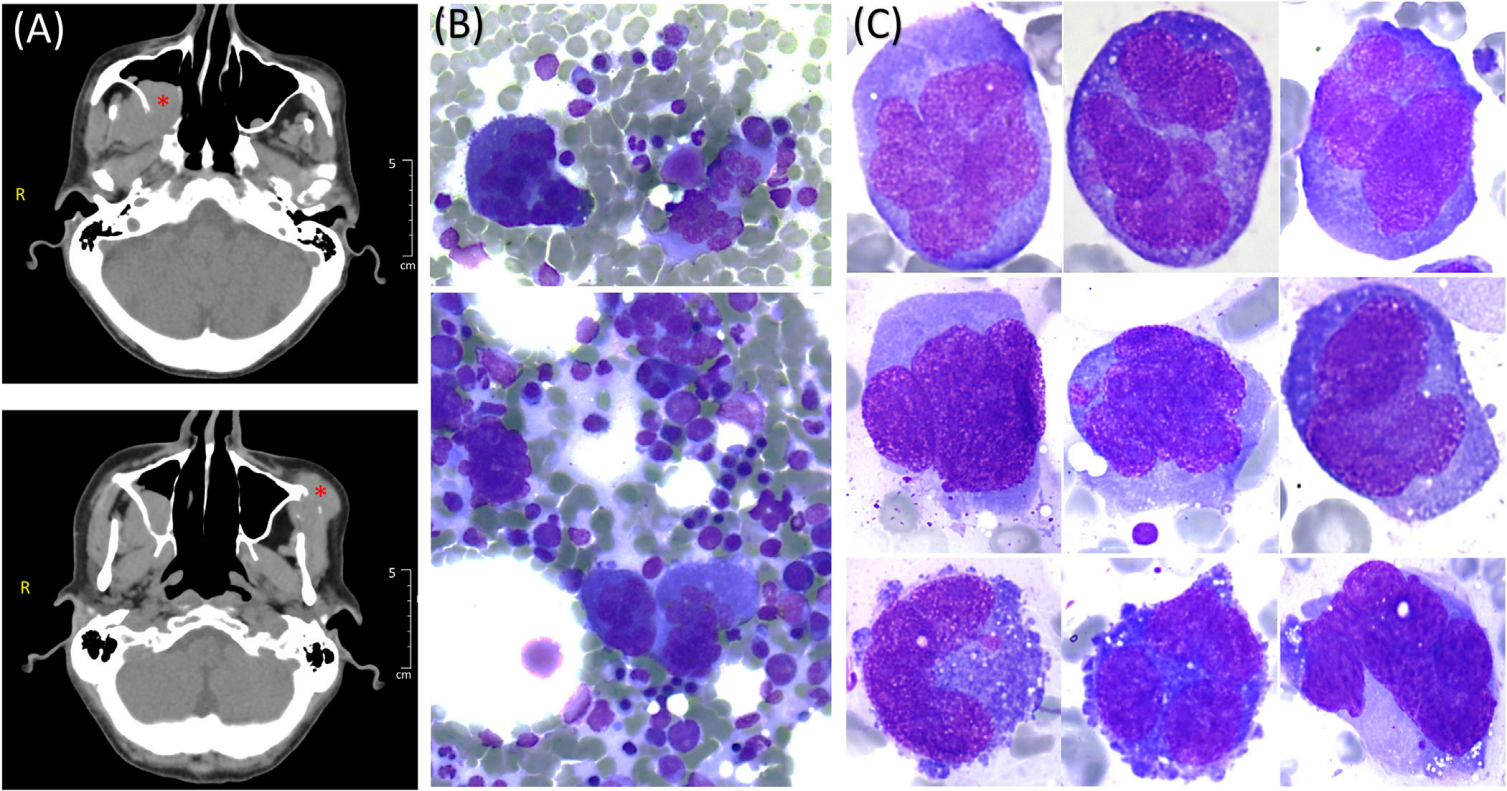
(A) Axial computed tomography of the head showed a mass in the right infratemporal fossa in the upper panel, while the lower panel revealed a mass in the left cheek causing minimal lysis of the left maxilla. (B and C) May‒Grunwald Giemsa stain, ×20 and ×100 objectives, respectively, demonstrating a variant of neoplastic plasma cells with megakaryocytoid features, characterized by markedly increased cell size.

Involvement of cerebrospinal fluid was absent. Bone marrow aspirate exhibited large to giant atypical cells (averaging 60‒70 µm in diameter) with a polymorphic nuclear pattern (abnormally lobated or multinucleated), prominent nucleoli, and abundant deeply bluish, occasionally vacuolated cytoplasm (Figure 1B,C). Flow cytometry of the bone marrow aspirate showed no conclusive results; however, the morphological aspect suggested a rare variant of neoplastic plasma cells—megakaryocytoid—where the cells exhibited markedly increased size, similar to that of a megakaryocyte.

Furthermore, the bone marrow biopsy confirmed the diagnosis of multiple myeloma, with immunohistochemistry demonstrating the presence of lambda monoclonal plasma cells (CD38+, CD138+, CD56‒, and CD117+). The left cheek mass was also found to be infiltrated by plasma cells, which exhibited the same megakaryocytoid morphology. Epstein‒Barr encoding region in situ hybridization was negative. No expression of LMP1 EBNA1 or EBNA2 was detected. HHV8 staining was negative. No biopsy of the cranial mass was performed.

Fluorescence in situ hybridization on selected plasma cells detected a gain of the *IgH* (14q32) locus with variant rearrangement as the sole anomaly. Three years post‐diagnosis, the patient shows myeloma progression and myeloma cast nephropathy despite partial response to multiple therapies, including ongoing treatment with carfilzomib, dexamethasone, and pomalidomide.

This case underscores the heterogeneous nature of neoplastic plasma cells, which can present with features resembling a wide range of hematologic and non‐hematologic disorders, thus posing significant diagnostic challenges [1]. In this case, the cells exhibit large, atypical morphology with markedly pleomorphic nuclei, resembling dysplastic megakaryocytes.

## Author Contributions

Radu Chiriac wrote the manuscript and conducted the cytological analysis. Zofia Gross followed the patient and supplied patient information. All authors contributed to the final manuscript.

## Ethics Statement

This manuscript respects the ethic policy of CHU Lyon for the treatment of human research participants.

## Patient Consent Statement

The authors have confirmed that a patient consent statement is not required for this submission, as no patient‐identifying data were used.

## Conflicts of Interest

The authors declare they have no conflicts of interest.

## Clinical Trial Registration

The authors have confirmed clinical trial registration is not needed for this submission.

## Permission To Reproduce Material From Other Sources

The authors declare no use of third‐party material in this study for which formal permission is required.

## Data Availability

Data sharing is not applicable to this article as no new data were created or analyzed in this study.
